# The Application of REDOR NMR to Understand the Conformation of Epothilone B

**DOI:** 10.3390/ijms18071472

**Published:** 2017-07-09

**Authors:** Jae-Ho Lee, Moon-Su Kim, Hyo Won Lee, Ihl-Young C. Lee, Hyun Kyoung Kim, Nam Doo Kim, SangGap Lee, Hwajeong Seo, Younkee Paik

**Affiliations:** 1 Department of Chemistry, Chungbuk National University, 1 Chungdae-ro, Cheongju, Chungbuk 28644, Korea; ljhlight@naver.com (J.-H.L.); anstnek0105@naver.com (M.-S.K.); 2 Drug Discovery Division, Korea Research Institute of Chemical Technology, 141 Gajeong-ro, Yuseong-gu, Daejeon 34114, Korea; iychoi50@gmail.com; 3 New Drug Development Center, Daegu-Gyeongbuk Medical Innovation Foundation, 88 Dongnae-ro, Dong-gu, Daegu 41061, Korea; hyunkyoungkim@dgmif.re.kr (H.K.K.); namdoo@gmail.com (N.D.K.); 4 Spin Physics & Engineering Team, Korea Basic Science Institute, 169-148 Gwahak-ro, Yuseong-gu, Daejeon 34133, Korea; sgl757@kbsi.re.kr; 5 Daegu Center, Korea Basic Science Institute, 80 Daehak-ro, Buk-gu, Daegu 41566, Korea; shj09301@hanmail.net

**Keywords:** microtubules, epothilone B, REDOR, solid-state NMR, bioactive conformation

## Abstract

The structural information of small therapeutic compounds complexed in biological matrices is important for drug developments. However, structural studies on ligands bound to such a large and dynamic system as microtubules are still challenging. This article reports an application of the solid-state NMR technique to investigating the bioactive conformation of epothilone B, a microtubule stabilizing agent, whose analog ixabepilone was approved by the U.S. Food and Drug Administration (FDA) as an anticancer drug. First, an analog of epothilone B was designed and successfully synthesized with deuterium and fluorine labels while keeping the high potency of the drug; Second, a lyophilization protocol was developed to enhance the low sensitivity of solid-state NMR; Third, molecular dynamics information of microtubule-bound epothilone B was revealed by high-resolution NMR spectra in comparison to the non-bound epothilone B; Last, information for the macrolide conformation of microtubule-bound epothilone B was obtained from rotational-echo double-resonance (REDOR) NMR data, suggesting the X-ray crystal structure of the ligand in the P450epoK complex as a possible candidate for the conformation. Our results are important as the first demonstration of using REDOR for studying epothilones.

## 1. Introduction

Solid-state NMR has provided powerful techniques to examine the molecular structures of biological system-bound drugs that pose a challenge to analysis by conventional methods such as X-ray crystallography and solution NMR spectroscopy [1,2]. Among these, rotational-echo double-resonance (REDOR) is a well-established technique used for precise measurements of interatomic distances by exploiting stable isotopes of the nuclei abundant in biological solids, such as ^13^C, ^15^N, and ^2^H to avoid structural modification [3]. Pharmaceutical compounds often contain fluorine and use of the high-γ nucleus ^19^F is helpful to enhance the sensitivity and range of measurable interatomic distances by compensating the low sensitivity and small range of dipolar interactions of other isotopes [4,5]. For example, REDOR was successfully applied to determine the conformation of microtubule-bound paclitaxel on a lyophilized sample of microtubules loaded with the fluorine-labeled analog of the ligand [6,7], which led to the synthesis and development of a novel compounds [8,9]. Recently, ^19^F NMR methodology for quantifying the binding affinity between proteins and low-complexity molecules was reported [10].

Among natural and synthetic epothilones, the epothilone B derivative ixabepilone (Ixempra^®^) was approved by the U.S. Food and Drug Administration (FDA) for the treatment of metastatic and advanced breast cancer [11]. Good water solubility [12], activity against multidrug-resistant cells [13], blood-brain barrier (BBB)-permeability [14], and biosynthesis [15] are the characteristics of epothilones better for cancer treatments in comparison to paclitaxel [16]. Structural studies on the conformations of tubulin-bound paclitaxel [8,9] and epothilone A [17,18,19,20,21] have provided a wealth of information to explain the structure–activity relationship (SAR) of these microtubule (MT)-stabilizing agents (MSAs) against various cell lines. In general, epothilone resistance was observed in cells with β-tubulin mutated at Ala231, Thr274, Arg282, or Gln292, and paclitaxel resistance in cells with β-tubulin mutated at Phe270 or Ala364 [22,23,24]. SAR studies have led to developments of several semi-synthetic derivatives of epothilones and purely-synthetic sagopilone as well [25,26].

**Figure 1:**
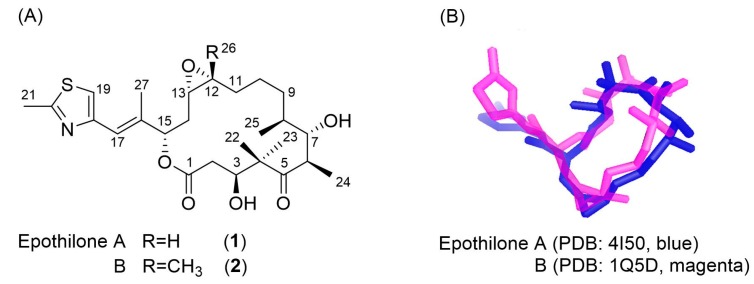
(**A**) The chemical structures of epothilone A and B; (**B**) The binding modes of epothilone A (PDB 4I50, blue) and B (PDB 1Q5D, magenta), the spatial coordinates being arbitrary and three-dimensionally rotated for the two conformations to overlap.

Epothilone A and B share a common chemical skeleton (Figure 1). However, their potency profiles are very different from each other, epothilone B shows cytotoxicities in vitro almost an order of magnitude higher than epothilone A [27]. On the other hand, the biological activity of epothilone D, which is lacking the characteristic epoxide group, is very close to epothilone B [28]. It is, therefore, important to elucidate the binding conformation of epothilone B in order to understand the origin of such a large difference, and for future drug developments, as well. In contrast to epothilone A, only a few structural studies have focused on the three-dimensional (3D) structure of tubulin- and/or microtubule-bound epothilone B [20,29,30]: an X-ray crystal structure was reported for epothilone B complexed to cytochrome P450epoK of *Sorangium cellulosum* [31]. A solution NMR study suggested a conformation of the tubulin-bound epothilone B that has much resemblance to the tubulin-bound epothilone A [20]. A solid-state NMR study on epothilone B-bound microtubules successfully found the ligand sites that were responsible for hydrogen bonding to tubulin [30], which was carried out on a frozen solution sample in lieu of powder. While electron microscopy (EM) was powerful enough to reveal the structure of tubulin-bound paclitaxel [8,9] and epothilone A [18,19,20,31], no systematic EM study has yet been reported for tubulin-bound epothilone B.

In this work, we synthesized a doubly-labeled analog of epothilone B: first, the dimethyl groups of C-4 in the macrolide were replaced with trideuteriomethyl groups for ^2^H-observed NMR to avoid any background signal problems. Second, a fluorine atom was introduced to C-26 methyl next to the epoxide by diethylaminosulfur trifluoride (DAST) fluorination of the corresponding 26-hydroxy compound, because several synthetic routes to 26-hydroxyepothilone B are available and 26-fluoroepothilone B shows a high potency against human cancer cell lines [32,33]. The doubly-labeled analog of epothilone B was bound to microtubules, then lyophilized into powders and subjected to solid-state NMR spectroscopy. Experimental REDOR data were analyzed using SIMPSON [34], compared to the structural models of epothilones in the literature [18,19,20,31], then used as guidance to searching and evaluating an X-ray crystal structure for the conformation model.

## 2. Results

### 2.1. Design of Epothilone B for Rotational-Echo Double-Resonance (REDOR)

**Figure 2:**
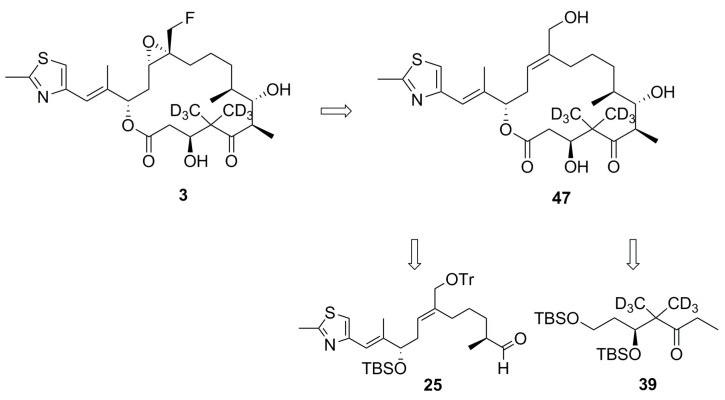
Retrosynthesis of analog **3**.

Although a trifluoromethyl group (–CF_3_) was widely used as the label for REDOR distance measurements [4,35], and for orientation-dependent NMR studies of membrane associated protein, as well [36,37], earlier work had shown that the labeling of fluorine atoms could affect the tubulin-binding affinity of paclitaxel by as much as two orders of magnitude [7]. A monofluoromethyl group (–CH_2_F) was also successfully employed as the REDOR label for measuring the relatively long intermolecular distances (~8 Å) of a membrane-bound antimicrobial peptide [38]. A limitation of using –CH_2_F group as a NMR probe for structural studies was suggested due mainly to the lack of its motional averaging, based on the segmental motions in amino acid crystals detected by ^2^H and ^19^F NMR at ambient temperature [36]. However, the molecular environments in biological solids are soft and flexible, being very different from those found in crystalline powders of amino acids where highly-charged and polar atomic networks can easily prevent the asymmetric –CH_2_F group from rotational motions. More importantly, the antitumor activities of synthetic 26-fluoroepothilone B were reported to be very similar to those of natural epothilone B in vitro [39] and in vivo [32]. Additionally, deuterium labeling of the two gem-methyl groups at C-4 would have negligible effects to any conformational changes of the macrolide and, thus, to its tubulin-binding affinity. Based on this analogy, an analog of epothilone B (**3**) was designed and chemically synthesized following the literature, with a few modifications for deuterium labeling (Figure 2) [40,41].

### 2.2. Cell Cytotocicity of Synthetic Epothilone B

The cytotoxicities of analog **3** on human lung cancer A549 and cervix cancer HeLa cells were measured as being close to those of natural epothilone B (Table 1). Significant increases (50~150 times) in the IC_50_ values were reported for epothilone B analogs with other chemical moieties at the C-26, such as –CHO and –CH_2_I [39]. Therefore, we reasonably assume that the conformation of microtubule-bound analog **3** represents the bioactive conformation of natural epothilone B.

### 2.3. The Deuterium Lineshape of Synthetic Epothilone B, Microtubule-Bound

**Figure 3:**
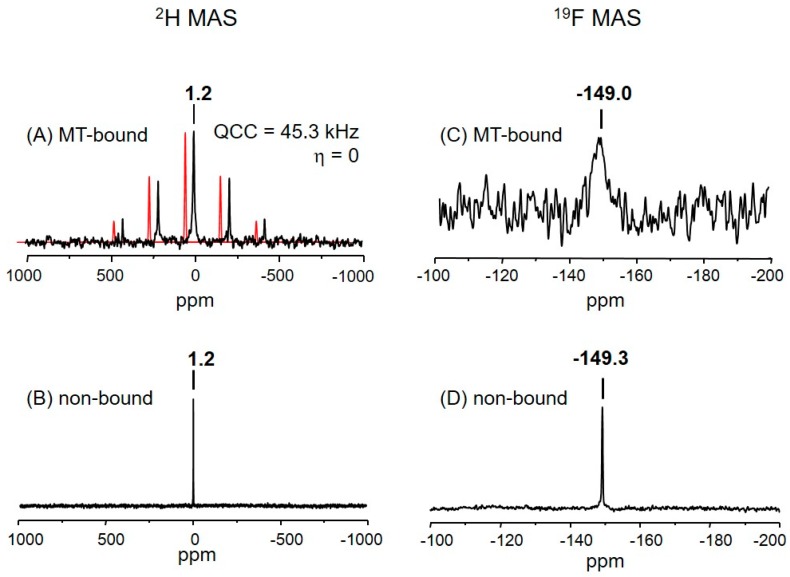
Solid-state magic-angle spinning (MAS) NMR spectra of analog **3**, microtubule-bound (**A**,**C**) or dispersed in PIPES buffer (**B**,**D**). (**A**) Experimental (black) and simulated (red; shifted left for clarity) ^2^H spectra with the calculated quadrupolar parameters displayed, 81,910 scans at 0.5 s repetition; (**B**) Experimental ^2^H spectrum; (**C**) Experimental ^19^F spectrum, 2560 scans at 50 s repetition; (**D**) Experimental ^19^F spectrum. All the spectra were acquired at ambient temperature with Hahn-Echo without proton decoupling and the isotropic resonances are marked on the spectra.

One isotropic resonance was observed at 1.2 (±0.5) ppm in the ^2^H magic-angle spinning (MAS) NMR spectrum of the microtubule-bound analog **3** (Figure 3A). The signal is from the C-22 and C-23 methyls of the analog **3**. Since the difference in the chemical shifts of the two methyl protons in epothilones is only about 0.3 ppm [12], deuterium signals of the two could hardly be distinguishable in the solid-state spectrum.

A deuterium quadrupolar (I = 1) pattern was obtained with at least four distinguished sidebands located at multiples of the sample spinning (13 kHz) from the isotropic resonance (Figure 3A). The MAS pattern is symmetric with respect to the central peak, a characteristic of deuterium lineshape for the rotating methyl groups [42]. Further analysis of this quadrupolar MAS pattern resulted in ~45.3 kHz for the size of the quadrupole coupling constant (QCC) (see Section 3). It is interesting to compare it with the ^2^H MAS spectrum in Figure 3B which was acquired from the analog **3** dispersed in the PIPES buffer in the absence of tubulin proteins and which gave rise to a single resonance at 1.2 ppm without any observable spinning sidebands (Figure 3B).

Since the symmetry axis of deuterium quadrupolar interaction is aligned along C–D bonds, ^2^H NMR lineshapes are sensitive to the geometry and motions of the deuterated segments [42]. The difference observed between the two ^2^H MAS NMR patterns (Figure 3A,B), which were acquired from the same methyls (C-22 and C-23) in analog **3**, can be ascribed to different molecular motions occurring in the two environments. The quadrupolar anisotropy (~45.3 kHz) in the ^2^H spectrum of microtubule-bound analog **3** (Figure 3A) may be an indication that the ligand molecule is in the bound state, the conformation of macrolide being static in the quadrupolar NMR timescale (τ_c_ > 22 µs). The residence time of tubulin-bound epothilone A in solution was estimated to be lot longer than 10 ms [17]. On the other hand, the macrolide of analog **3** in the PIPES solid buffer matrix (Figure 3B) seems to change its conformation very rapidly to the degree of almost free isotropic molecular motions which average out the anisotropy of ^2^H quadrupolar interaction, resulting in a single ^2^H resonance without any sidebands.

### 2.4. The Fluorine Signal of Synthetic Epothilone B, Microtubule-Bound

Figure 3C shows the ^19^F MAS NMR spectrum of the microtubule-bound analog **3** which gave rise to one isotropic resonance at −149.0 ppm with no observable sidebands. A part of the reason for the low signal-to-noise ratio is because the spectrum was acquired without proton decoupling, even though the spectral data were collected for about 36 h, on top of the very low concentration (<1 wt %, 0.18 mg) of the active ligand in the powder sample (see Table S2 for sample composition). It is, again, interesting to compare it to the ^19^F spectrum (Figure 3D) acquired from the analog **3** dispersed in PIPES buffer in the absence of tubulin proteins with a high signal-to-noise ratio. ^19^F chemical shifts of the two signals are almost identical, which may be an indication that dissociation of the epoxide (–C12–O–C13–; Figure 1A) ring of analog **3**, where the 26-fluoromethyl is attached, did not occur after binding to microtubules. This may provide a new approach to a more fundamental understanding on the findings that “the replacement of one hydrogen atom of C-26 methyl group by relatively small and apolar substituents such as F, Cl, CH_3_, or C_2_H_5_, produces analogs which are only slightly less potent in vitro than Epo B” [43]: i.e., it is possible that the epoxide structure is tolerated during the MT-binding process when the substituent at C-26 methyl is small and apolar.

On the other hand, the linewidths of two ^19^F signals (Figure 3C,D), both from the 26-fluoromethyl of analog **3**, are very different from each other, 5.5 vs. 0.5 ppm. The substantially broader linewidth (5.5 ppm) may be another piece of evidence for the characteristic molecular dynamics of microtubule-bound analog **3** in comparison to the non-bound (see Section 3).

### 2.5. Using a Double Resonance Spectrometer for ^2^H{^19^F} REDOR

**Figure 4:**
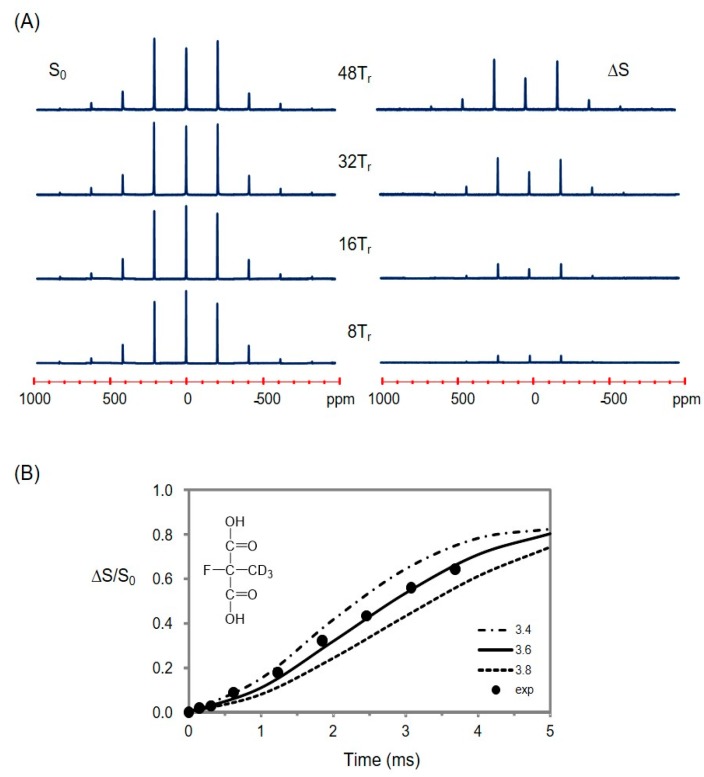
(**A**) The ^2^H{^19^F} REDOR spectra (*S*_0_ and Δ*S*) of 2-fluoro-2-methyl-d_3_-malonic acid ([2-F,2-Me-d_3_]MA) with the dephasing times marked on the corresponding spectra; (**B**) ^2^H{^19^F} REDOR curves (Δ*S*/*S*_0_), where the experimental dephasing (•) is consistent with the SIMPSON calculation for a single distance of 3.6 (±0.2) Å. The NMR spectra were acquired at ambient temperature.

Though the high-γ of ^19^F is helpful to enhance the NMR sensitivity and increase the range of measurable distances by REDOR, its proximity to ^1^H resonance frequency often makes it difficult to observe fluorine while performing proton decoupling with a conventional spectrometer. A strategy of performing REDOR experiments with a two-channel spectrometer for biological solids was previously developed for a ^2^H-^19^F spin pair [4]: deuterium (X) was observed without ^1^H decoupling as the isotope has no directly bonded protons and possesses comparatively weak dipolar couplings to protons, while the high-frequency channel (H/F) of the conventional double resonance spectrometer was used for ^19^F-spin inversion. First, we used a synthetic compound, 2-fluoro-2-methyl-d_3_-malonic acid ([2-F,2-Me-d_3_]MA), and its intramolecular ^2^H-^19^F spin pair (d ~ 3.6 Å) as a reference model for testing the feasibility of implementing ^2^H{^19^F} REDOR to our 400 MHz wide-bore spectrometer equipped with only two-channel hardware, H/F and X. By employing a pulse sequence shown in Figure S3 [4,7], distortion-free full-echo deuterium signals (*S*_0_) and difference signals (Δ*S*) were successfully acquired without proton decoupling (Figure 4A). SIMPSON analysis for the signals successfully reproduced a single distance of 3.6 (±0.2) Å for the spin pair (Figure 4B).

### 2.6. Information for the Microtubule-Bound Epothilone B Conformation

**Figure 5:**
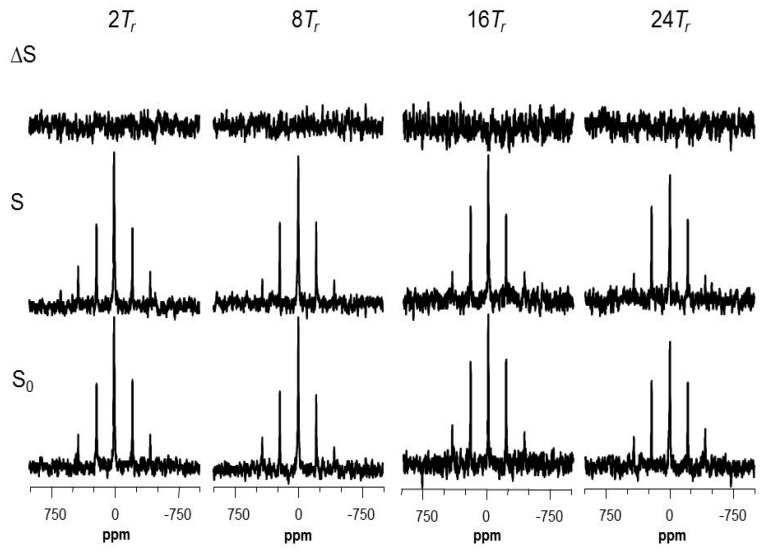
^2^H{^19^F} REDOR spectra of the microtubule-bound analog **3** as a function of dipolar evolution with magic-angle spinning at 13,000 Hz, full-echo (*S*_0_), dephased-echo (*S*), and difference (Δ*S*) spectra. Dipolar evolution times are marked in multiples of the rotor period (*T_r_*). The acquisition scans for the 2, 8, 16, and 24*T_r_* experiments were 81,920, 163,840, 163,840, and 368,640, respectively. The NMR experiments were performed at ambient temperature.

**Figure 6:**
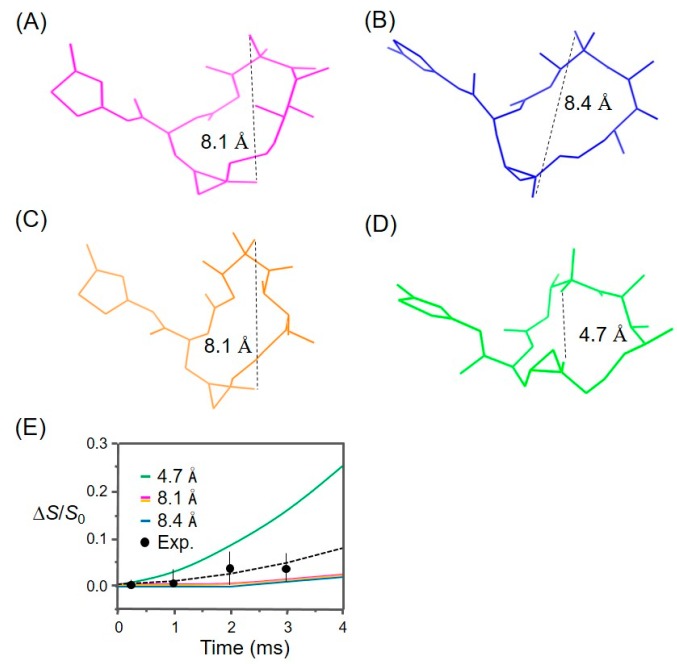
Comparison of the experimental REDOR data with protein-bound conformations of epothilones in the literature. (**A**) Cytochrome P450epoK-bound epothilone B (PDB 1Q5D); (**B**) T_2_R-TTL-EpoA complex (PDB 4I50); (**C**) Tubulin-bound epothilone A [20]; (**D**) Tubulin-bound epothilone A in zinc-stabilized 2D sheets (PDB 1TVK); and (**E**) The SIMPSON curves of ^2^H{^19^F} REDOR dephasing calculated as a function of the dipolar evolution time for the above four conformers. For a systematic comparison, 26-methyl was added to the known structures of epothilone A (**B**–**D**). A shorter distance between the 26-fluoromethyl to the germinal trideuteriomethyl groups (C-22 and C-23) is displayed by dotted lines with the marked value. The error bars shown in the difference (Δ*S*) spectra (**E**) represent one standard deviation of the noise levels from the peak maxima.

Thereafter, symmetric full-echo (*S*_0_), dephased (*S*), and difference (Δ*S*) ^2^H{^19^F} REDOR spectra were successfully acquired from the microtubule-bound analog **3** (Figure 5). However, only small dephasings (≤0.04) of the ^2^H signals were detected after up to 24 rotor periods (Figure S5). Nonetheless, a few possible REDOR distance pairs (^2^H-^19^F) were suggested between the deuterium and fluorine labels by fitting the available experimental data with SIMPSON calculations, because the distances between fluorine (C-26) and two trideuteriomethyls (C-22 and C-23) may vary depending on the orientation of the two methyl groups towards the fluorine atom (Table S2; Figure S6). Although the analytical results were not sufficient to determine the relevant intramolecular distances, it can provide a criterion to compare various structural models for the bioactive conformation of epothilone B (Figure 6; see Section 3). The REDOR analysis suggested at least 5.0 Å or longer for the distances between the fluorine and deuterium labels.

## 3. Discussion

Clinical studies of epothilones are widely conducted for curing brain tumors and axonal damage, and especially for taxane-resistant tumors [14,25]. Human non-small cell lung (NSCL) cancers—when single-point mutations occurred in β-tubulin at residues Gln292, Pro173, or Tyr422—showed an increase in epothilone-resistances by 10~100 times while having an order of higher drug sensitivities to epothilone B than A [44]. The difference in drug sensitivities increased even more if the mutation had occurred at Thr274 or Arg281 (β-tubulin) in human ovarian cancer cells [22]. Structural studies using an electron microscopy [8,18], X-ray [19], and NMR [20] have found that these residues are the ones located near the drug binding site and form direct molecular contacts with the binding drug. However, most of those structural studies are based on experimental data collected from tubulin dimers, oligomers, or 2D sheets. Therefore, a breakthrough in future drug development probably requires some more experimental data collected directly from the epothilones in action, i.e., in the microtubule-bound state.

### 3.1. Analysis of the REDOR Data for Epothilone B Conformation

Figure 6 shows the representative conformations of tubulin-bound epothilones reported in the literature. Our experimental REDOR data (Δ*S*/*S*_0_) were compared with the SIMPSON curves calculated for the corresponding ^2^H-^19^F spin pairs in each structure, of which only the shorter distances were depicted by dotted lines. The comparison likely indicates that the experimental data deviates from the curve calculated for the conformation in Figure 6D, while being consistent with the other three structures, which are very similar in the relevant distances (Figure 6A–C). This suggests that the macrolide of microtubule-bound epothilone B may have a different conformation from the one of tubulin-bound epothilone A in tubulin 2D sheets [18]. SIMPSON calculations assumed that the observed nucleus (^2^H) is located at the center of three deuterium atoms, the distance between the two CD_3_-groups being fixed at 2.7 Å. Additionally, it is also assumed that the source of dephasing ^19^F is located near the center of H_2_F triangle in CH_2_F-group, as the –CD_3_ and –CH_2_F undergoing fast C_3_-type rotations. These assumptions are used in common in the literature [38,42], and are also consistent with the molecular dynamics information obtained from our ^2^H and ^19^F signals (see below).

Since the experimental REDOR data did not deviate from the SIMPSON curve calculated for the conformation of epothilone B found in the X-ray crystal structure of the P450epoK complex (Figure 6A), the only crystal structure reported so far of epothilone B in action, we went further to evaluate a docking behavior of the structure with a tubulin dimer (see below).

### 3.2. Evaluation of an X-ray Crystal Structure (PDB 1Q5D) for the Epothilone B Conformation

**Figure 7:**
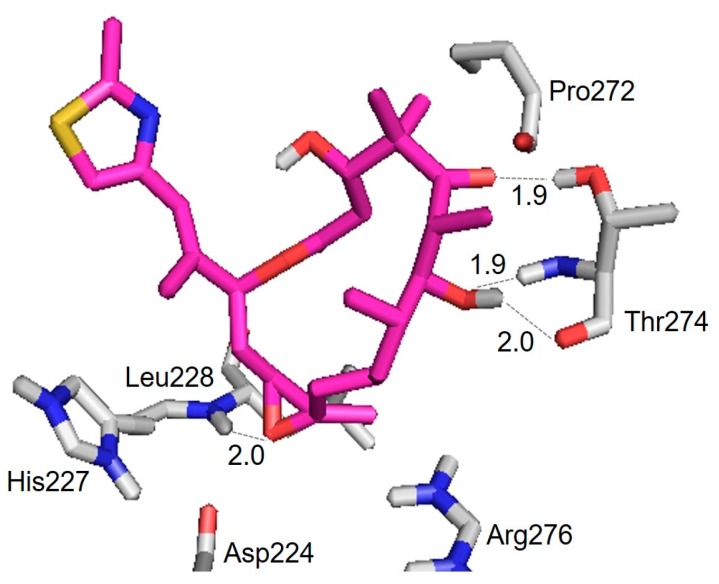
Hydrogen bond interactions of the epothilone B conformer epoB(TUB)_1q5d with amino residues in tubulin dimer. Oxygen, nitrogen, sulfur, and hydrogen are indicated in red, blue, yellow, and white, respectively. Carbon in the drug molecule and tubulin protein are indicated magenta and grey, respectively. Hydrogen bonds are indicated by dotted lines, and their lengths are given.

Figure 7 shows the structure of an epothilone B conformer epoB(TUB)_1q5d docked to a tubulin dimer (binding energy ≅ −7.91 kcal/mol) and the hydrogen bond interactions with the amino residues in β-tubulin (Table 2). Its binding mode is somewhat different from the ones generally found for epothilone A conformers in the literature [19,20]: the polar atoms O(5) and O(7), in lieu of O(1), made hydrogen bonding to OH(Thr274) and NH(Thr274), respectively, in the *M*-loop. The polar atom O(epoxide), in lieu of O(7), contacted NH(Leu228) of the *H7* helix. However, when another docking experiment was carried out for epothilone A, all of the important hydrogen-bonding contacts were reproduced between the polar atoms of epothilone A and the amino residues of β-tubulin at a low binding energy (−6.11 kcal/mol) (Figure S7) [19]: i.e., residues in the *M*-loop of β-tubulin formed hydrogen bonding with the ligand between O(1) and NH(Thr276) at 2.0 Å, and O(3)H and NH(Gln281) at 2.1 Å. The carbonyl (C=O) of Asp226 in the *H7* helix made a contact to O(7)H at 1.9 Å. A molecular modeling study on the epothilone B/tubulin interaction was reported by adopting an initial conformation obtained from the single crystal X-ray structure of pure epothilone B [22]: two important hydrogen bondings were found between Leu228(NH) and O, and Thr274(NH) and O(7), which is consistent with our results (Table 2).

The hydroxyl O(3)H of epothilone A is in general considered to be important for high potency of epothilones. However, a few analogs of epothilone without O(3)H have been synthesized and showed high potencies [45]. Thus, it is possible that the 3-hydroxyl group may not be directly involved in the intermolecular interactions with β-tubulin. The docking energy resulting from EpoB(TUB)_1q5d is comparable to the one (−8.2 kcal/mol) reported for epothilone A in a similar study [46]. Therefore, based on the REDOR experimental data and the evidences from docking evaluation, we suggest epoB(TUB)_1q5d, derived from the X-ray crystal structure of epothilone B in cytochrome P450epoK complex [31], as a potent model for the conformation of microtubule-bound epothilone B.

### 3.3. Applicability to Other Epothilone Derivatives

The isotope-labeling scheme used here for the synthesis of analog **3** cannot be applied to epothilone A, as this compound lack the 26-methyl group for labeling with fluorine. Therefore, another scheme should be designed for a REDOR study of epothilone A.

On the other hand, the labeling scheme may readily be adopted for studying the MT-bound conformation of ixabepilone in the above manner. Ixabepilone (aza-epothilone B) was developed by Bristol-Myers Squibb (BMS, New York, USA) to improve the metabolic stability and safety profile of epothilone B, for which the lactone oxygen of epothilone B was replaced by nitrogen resulting in a higher water stability and a lower tubulin-binding affinity [43]. The two drugs showed very similar profiles in the antitumor activity against taxane-resistant cells [47], and molecular interaction with tubulin isotypes, as well [48]. However, very few structural studies were reported for the binding mechanism and bioactive structure of ixabepilone, while numerous such studies are available for epothilone B. A computational study reported recently that the two compounds have different conformational preferences in vacuo [49]: epothilone B adopts only the exo-conformation, while ixabepilone exists as a mixture of the exo and endo.

Our labeling scheme can also be used to investigate the microtubule-bound conformation of epothilone D (deoxy-epothilone B). Epothilone D is another potent agent possessing a chemical structure somewhat different from epothilone B, where the characteristic epoxide (–C12–O–C13–) group was replaced by an olefin (–C=C–) group. Nonetheless, its biological profiles are very close to those of epothilone B [28]. Interestingly, the crystal structures and interaction environments found in P450epoK complex are almost identical for the two compounds, except that one more hydrogen-bonding interaction is formed for epothilone B between its epoxide oxygen and a water ligand of the enzyme [31]. It was also reported that the 26-fluoro analog of epothilone D showed high potencies against human carcinoma [39]. Recent studies showed that epothilone D is a promising drug for treatments of traumatic brain injury (TBI) by promoting the regeneration of neuronal sprouts [25].

### 3.4. Molecular Motions of the Microtubule-Bound Epothilone B

#### 3.4.1. Indications for the Binding

The large QCC (~180 kHz) of C–D bond gives rise to specific ^2^H NMR lineshapes representing the frequency, type, amplitude, and geometry of motions involving the bond [42]. Analysis of the ^2^H MAS pattern (Figure 3A) produced a QCC of ~45.3 kHz, being smaller by ca. 19% than the size of QCC (≈56 kHz) expected for rotating methyl groups in organic solids. Comparison with the ^2^H spectrum in Figure 3B clearly shows that the microtubule-bound epothilone B should no undergo isotropic random motions, i.e., such as translations or rotations in the binding pocket faster than the quadrupolar NMR timescale. Instead, the tetrahedral geometry at C-4, where the dimethyls were attached, can undergo torsional motions with a small amplitude at the ambient temperature. Hence, the apparent reduction in ^2^H QCC is possibly due to further averaging effects by small amplitude motions, such as rocking and wagging of the the C4–C22 and C4–C23 bond axis within a cone angle of ca. 10.5°, i.e., an order parameter (3cos^2^ϕ − 1) of 0.81 with ϕ = 10.5° [42]. Local motions of dideuteriomethylene in long aliphatic carbon chains, occuring at low temperatures when chain rotaions are frozen, are a well-known example of ^2^H NMR lineshape studies on such motions [50]. However, effects of these motions on the REDOR analysis would be within the experimental errors (±0.2 Å) given the long distances to be measured [38,51]. 

#### 3.4.2. The Fraction of Non-Bound Analog **3**

It is possible that some of non-bound ligands might have remained during the sample preparation procedures including pelleting of drug-bound microtubules [52], confusing a direct interpretation of the signals in the NMR spectra. For example, a slightly better fitting profile was obtained for the experimental ^2^H MAS powder pattern when the spectrum was simulated with two spectral components: one from the microtubule-bound ligand (91%; QCC = 49.1 kHz) and the other from free ligand (9%; QCC ≈ 0). Therefore, it is possible that a maximum of 9% ^2^H signals might have come from the non-bound drug. However, considering the low concentration (20 µM) of the analog **3** dissolved in the original polymerizing buffer (see Section 4), the remnant portion of non-bound ligand entrapped in the microtubule pellet (≈100 µL) during the centrifuge step, and survived through additional four-times washing it with fresh buffer, is estimated to be too small (≤1%) to affect the MAS pattern. Moreover, the difference (Δ*S*/*S*_0_) signal used for REDOR analysis included only the four major sidebands but excluded the central peak (Figure 4). Therefore, a fraction of non-bound analog **3**, if any, would have negligible effects to our NMR experiments.

#### 3.4.3. The Fluorine Linewidth

The ^19^F spin-lattice relaxation times (T_1_s) of monofluoromethyl of aliphatic amino acid were reported in a range of 0.3~20 s [36]. Since fluorine is used only as a dipolar source for dephasing the deuterium signals in our ^2^H-observed, ^19^F-dephased REDOR experiments, the broad linewidth (i.e., short T_2_) of the ^19^F signal observed in Figure 3C would not be a problem for REDOR data analysis if the ^19^F T_1_ were long enough to perform REDOR experiments (≥0.1 s).

On the other hand, it is necessary to analyze the ^19^F signal linewidth to find out whether or not the –CH_2_F group in the microtubule-bound ligand undergoes methyl rotations, an issue to be resolved for REDOR data analysis. Since the 26-fluoromethyl is attached to C-13 in the macrolide, nine C–C bonds away from the ^2^H NMR sources (C-22 and C-23), analysis of the ^19^F signal can also provide additional information on the binding stability of the microtubule-bound epothilone B as below.

The ^19^F chemical shift anisotropies (CSAs) of CH_2_F-group of aliphatic amino acids are in a range of 10–30 ppm [36]. These relatively small CSAs can be effectively averaged out by the sample spinning condition (13 kHz) used here. Therefore, dipolar couplings between the fluorine nuclei and neighboring protons must be the dominant contributors to the ^19^F linewidth (~5.5 ppm) observed in Figure 3, in particular, as the spectrum was acquired without proton decoupling. Direct dipolar couplings between the fluorine and the two protons within the CH_2_F-group are about 25 kHz (or ~66 ppm at 376.44 MHz). The sample spinning at 13 kHz must have averaged out most of these heteronuclear dipolar couplings down to 5.5 ppm. However, the ^19^F MAS NMR spectrum acquired from a sample [2-F,2-Me-d_3_]MA at the same sample spinning showed an even broader linewidth (8.5 ppm), although fluorine in the compound has no dipolar-coupled protons as close (~1.7 Å) to those in the CH_2_F-group (Figure S4). Therefore, an additional averaging process other than MAS should be working for the CH_2_F-group in MT-bound analog **3**. Motional averaging by a C_3_-type rotation is firstly considered, because the fluorine in [2-F,2-Me-d_3_]MA is attached to the quaternary carbon where such a process is impossible. On the other hand, the ^19^F MAS NMR spectrum acquired from the same 26-fluoromethyl of analog **3**, dispersed in PIPES buffer with no tubulin proteins (Figure 3B), gave rise to a much narrower linewidth (0.5 ppm), a flexible organic system where isotropic, random-motional averaging is possible at the fluorine site. In short, a comparison of the ^19^F linewidth (5.5 ppm) found in MT-bound analog **3** (Figure 3C) with those found in non-bound analog 3 (Figure 3D) (0.5 ppm) and [2-F,2-Me-d_3_]MA (Figure S4) (8.5 ppm) suggests that the 26-fluoromethyl of the MT-bound analog **3** undergoes C3-type rotational motions, but is far from isotropic, random motions.

## 4. Materials and Methods

### 4.1. Synthesis of Analog ***3***

The retrosynthesis of analog **3** is shown in Figure 2. Synthetic procedures and the spectral data of selected intermediate compounds were given as Supporting Information (Figure S1). The details of synthesis were reported elsewhere [53]. Compound **25** was synthesized from the Wittig reaction between compound **11** and **28** followed by successive reactions as displayed in Figure S1A. Compound **11** was from a starting material, (*S*)-malic acid, whereas the synthesis of compound **20** was started from methyl (*R*)-(−)-3-hydroxy-2-methylpropionate (Figure S1C). The ketoaldehydic compound **33** was initiated from diethyl malonate **26** as a starting material through a series of chemical transformations, including dimethylation with CD_3_I (Figure S1D). The Samarium-mediated Reformatsky reaction upon the chiral oxazolidinone derivative from **33** provided β-hydroxyketone **35**. The reduction, and subsequent rendering, of the resulting two hydroxyl groups as TBS ethers yielded compound **39** (Figure S1B). The two halves of the northern and southern units were coupled at the C-6 and C-7 positions via the aldol reaction. Then, the Yamaguchi lactonization was used for the formation of a macrolide backbone and the removal of protecting groups to give compound **47** (Figure S1E). The Sharpless Asymmetric Epoxidation (SAE), followed by fluorination with diethylaminosulfur trifluoride (DAST), eventually resulted in compound **3** (Figure S1F).

### 4.2. Cell Cytotoxicity (IC_50_) Test

Cell viability assay was conducted for the cytotoxicities of the labeled and natural epothilone B. A549 and HeLa cells were seeded in a 96 well plate at densities of approximately 3000–4000 cells per well. Cells were allowed to settle for 24 h prior to addition of drug. Drugs were added to the first well of the plate and serial diluted (1 to 3 nmol/L) to subsequent wells and incubated for 72 h. The medium was discarded and 100 µL of a 20% MTS solution (CellTiter 96 Aqueous One Solution; Promega Korea, Ltd., Seoul, Korea) was added to each well and then incubated for 1 h at 37 °C in a 5% CO_2_ incubator. Absorbance was measured at 490 nm. The experiments were carried out three times for each of the tested drugs. Each run entailed 9 or 10 concentrations (Figure S8).

### 4.3. Sample Preparations

A microtubule powder sample for REDOR experiments was prepared as reported previously [52]. In brief, 40 mg of purified porcine tubulin was polymerized in a Falcon 50 mL conical tube at 37 °C using 20 mL of tubulin assembly buffer containing 80 mM PIPES, 2 mM MgCl_2_, and 0.5 mM EGTA at pH 7.0 by successive addition of the labeled epothilone B to the concentration of 0.2, 2.0, and 20 µM at 5–10 min intervals. The final solution was incubated further for 1 h at 37 °C to obtain a suspension of microtubules, then was centrifuged on 30% glycerol cushion buffer using an Optima L-100 XP ultracentrifuge with a SW 41 Ti rotor for 20 min at 41,000 rpm at 37 °C. The supernatant was discarded. The pellet was rinsed four times with a fresh assembly buffer, snap frozen, and then lyophilized under low pressure (≤10^−4^ atm). The lyophilizing buffer was 10% sucrose in deionized water. Repeated trials have succeeded in increasing the content of the protein with respect to sucrose buffer in the lyophilized powder to 40 wt % while preserving the morphology of microtubules (Figure S2). For testing the REDOR sequence, a control sample was prepared by dissolving a synthetic model compound, 2-fluoro-2-methyl-d_3_-malonic acid, in PIPES buffer, which was snap frozen and then lyophilized. The constituents in the lyophilized powders are listed in Table S1, where it was assumed that a maximum of 50 µL of tubulin assembly buffer was entrapped in the microtubule pellet at the centrifuge step [52]. The difference between the weights of the lyophilized powder and the total sum of added components was regarded as the contribution from bound water molecules due to the highly-hygroscopic property of the sucrose network.

### 4.4. REDOR NMR Experiments

Solid-state REDOR NMR experiments were performed on a Bruker Avance II 400 MHz spectrometer using a double-resonance probe equipped with a 4-mm rotor spinning module at 13 kHz. ^2^H and ^19^F MAS NMR spectra were obtained using Hahn-Echo pulse sequence at resonance frequencies of 61.42 and 376.44 MHz, and were referenced to D_2_O (4.8 ppm) and CF_3_COOH (0 ppm), respectively. The 90° pulse lengths for ^2^H and ^19^F were 3.2 and 3.0 µs, respectively. The schematic pulse sequence of ^2^H{^19^F} REDOR for 4-*T_r_* dephasing signal (*S*) is shown in Figure S3A. Full-echo signal (*S*_0_) was acquired without the dephasing pulses on the ^19^F channel. The *xy-8* phase cycling for dephasing pulses was used. The REDOR sequences were carried out without employing proton decoupling as suggested by Grage et al. [4]. Internuclear distances were calculated from the REDOR dephasing signals by spectral simulations performed using SIMPSON [34]. SIMPSON simulations were carried out on the pulse sequence shown in Figure S3A up to 64 *T_r_*s using an ideal pulse for ^2^H and 100-kHz for ^19^F channels at 13 kHz spinning with 320 crystal orientations, with relevant NMR parameters: CSA(^19^F) = 20 ppm, QCC(^2^H) = 45.3 kHz. The size of CSA(^19^F) was adopted from the literature [36], and proton dipolar interactions were not included in the simulation as the effects were reported to be negligible in SIMPSON studies of a similar spin system [4]. For REDOR experiments, ca. 122 mg of a sample for analog **3**-bound microtubules and 90 mg of a sample for [2-F,2-Me-d_3_]MA were packed into 4-mm rotors for MAS, respectively; the former sample was transferred to the rotor using a home-built sampling tool [54] in order to keep the highly-hygroscopic sucrose buffer from moisture.

### 4.5. AutoDock Calculations

Docking calculations were carried out for epothilone B conformations using AutoDock software [55]. The structure of epothilone B in the cytochrome P450epoK complex (PDB 1Q5D) [31] was extracted, termed epoB(TUB)_1q5d, and then manually docked into the structure of tubulin-dimer in PDB 1TVK. The docking procedure was performed following the literature [46]. In brief, the structures were parameterized with Gasteiger atomic partial charges. A Lamarckian genetic algorithm was used using a cubic grid box with a spacing of 0.375 Å and dimensions of 22 Å with 2.5 × 10^7^ energy evaluations for each of 200 docking trials. For flexible dockings, two single bonds in the thiazole sidechain, C15–C16 and C17–C18, and all C–OH bonds of the ligand were set as rotatable. Additionally, four amino acids in β-tubulin, His227, Arg276, Arg282, and Thr274, which were known to interact strongly with the ligand were set as flexible. The conformations of macrolide were maintained as rigid. In order to check the validity of the above docking procedures, an additional experiment was carried out for the structure model of tubulin-bound epothilone A by Prota et al. [19], termed epoA(TUB)_4i50, for which the tubulin-dimer C and D chains in PDB 4I50 were utilized. Two more residues, Asp226 and Gln281, in β-tubulin were set as flexible for epoA(TUB)_4i50 in addition to the four residues, His229, Arg278, Arg284, and Thr276. The 3D conformations of the above two ligands were comparatively displayed in Figure 1B.

## 5. Conclusions

We have demonstrated the feasibility of using a REDOR NMR technique to investigate the bioactive structure of microtubule-bound epothilone B. A doubly isotope-labeled analog of epothilone B was successfully synthesized with its cytotoxic profile being measured to be close to the natural epothilone B. The size of deuterium quadrupolar parameter (QCC ≈ 45.3 kHz) observed in the MAS powder NMR pattern of microtubule-bound analog **3**, indicated that the ligand is in the bound state (τ_c_ > 22 µs). A non-variant ^19^F chemical shifts were observed for the analog before and after the microtubule-binding, evidencing that the epoxide-ring structure of epothilone B, to which the 26-fluoromethyl was attached, is tolerated during the MT-binding process in vitro. Symmetric ^2^H{^19^F} REDOR spectra were successfully acquired from the microtubule-bound epothilone B. The experimental data was lacking a precision to determine the relevant intramolecular distance, but provided a criterion (>5.0 Å) to compare various conformation models suggested for bioactive epothilone B structure. A docking evaluation was carried out for the X-ray crystal structure of epothilone B reported earlier in the P450epoK complex, because the macrolide conformation of the structure is consistent with our REDOR analysis. The solid-state NMR methodology developed here may open up new avenues for elucidating the bioactive structures of epothilone B, and other epothilone derivatives, in such a dynamic system as microtubules hardly accessible to conventional X-ray crystallography and solution NMR spectroscopy.

## Figures and Tables

**Table 1:** Human cancer cell growth inhibition (IC_50_; nmol/L).

Cell Line	A549	HeLa
Epo B (**2**)	127	41
Analog (**3**)	163	67

**Table 2:** Hydrogen bond parameters for the docked epothilone B conformer, epoB(TUB)_1q5d.

Hydrogen Bond	Distance (Å)
Thr274(CO) ⋯ HO(7)	2.0
Thr274(NH) ⋯ O(7)	1.9
Thr274(OH) ⋯ O(5)	1.9
Leu228(NH) ⋯ O	2.0

## Supplementary Materials

Supplementary materials can be found at www.mdpi.com/1422-0067/18/7/1472/s1.

## Abbreviations

[2-F,2-Me-d_3_]MA	2-fluroro-2-methyl-d_3_-malonic acid
MAS	Magic angle spinning
MT	Microtubule
NSCL	Non-small cell lung
QCC	Quadrupole coupling constant
REDOR	Rotational-echo double-resonance
SAR	Structure-activity relationship
