# Supplementary material for: The Application of REDOR NMR to Understand the Conformation of Epothilone B
Source: Int J Mol Sci. 2017 Jul 9;18(7):1472. doi: 10.3390/ijms18071472 (PMC5535963; doi:10.3390/ijms18071472)

Supporting Information

The Application of REDOR NMR to Understand the Conformation of Epothilone B

Spectral data for compounds **3, 7-10, 17, 18, 21, 23-25, 27, 29, 31, 32, 35-42, 45-48.**

Figure *S*1. Synthesis of analog **3** and its intermediate compounds: (A) Synthesis of compound **25**, (B) Synthesis of compound **39**, (C) Synthesis of compound **11**, (D) Synthesis of compound **33**, (E) Synthesis of compound **47**, (F) Synthesis of compound **3**, (E) Synthesis of compound **20**.

Figure *S*2. TEM images of the analog **3**-bound microtubules. The scale bars represent 1 μm (A) and 500 nm (B). Before collecting the TEM images, the lyophilized powder was resuspended in PEM buffer supplemented with 20 μM epothilone B.

Figure *S*3. A schematic representation of the $^{2}H\{^{19}F\}$ REDOR sequence for a dephasing signal (*S*) at $4T_r$.

Figure *S*4. Solid-state ^{19}F MAS NMR spectra of 2-fluoro-2-methyl-d_3-malonic acid ([2-F,2-Me-d_3]MA) lyophilized in pipes buffer.

Figure *S*5. The full-echo (S_0) and dephasing (*S*) spectra obtained by co-adding the most intense four spinning sidebands in the $^{2}H\{^{19}F\}$ REDOR spectra (Figure 5). The peak heights of the difference (ΔS) spectra and noise levels in parenthesis being marked on each spectrum in fractions of the corresponding full-echo spectra. The dipolar evolution times are marked on top in multiples of the rotor period (T_r).

Figure *S*6. $^{2}H\{^{19}F\}$ REDOR dephasing curves for possible distance pairs (see also Table *S2*).

Figure *S*7. Hydrogen-bond interactions of polar atoms of the epothilone A conformer *epoA(TUB)_4i50* with amino residues in tubulin dimer.

Figure *S*8. The cell viability assay curves for the [D,F]-labelled and natural epothilone B against human lung carcinoma (A549) and cervix adenocarcinoma (HeLa).

Table *S*1. Constituents of the lyophilized powders for REDOR NMR

Compound[a]	Analog **3**-bound microtubules		$[2\text{-F},2\text{-Me-d}_3]$MA	
	mg	wt%	mg	wt%
Sucrose	60	59	-	-
Tubulin	40	39	-	-
Labeled compound[b]	0.18	< 1	9.5	11
Pipes	1.21[c]	1	67.5	75
EGTA	0.02[c]	< 1	-	-
$MgCl_2$	0.01[c]	< 1	-	-
K^+ ion	0.23[c]	< 1	13[e]	14
Water	20[d]	17	-	-
Sum	122	100	90	100

[a]The numbers are estimated values based on the reaction conditions.

[b]The labeled compound is either analog **3** or $[2\text{-F},2\text{-Me-d}_3]$MA.

[c]From tubulin assembly buffer entrapped in the microtubule pellet (see text).

[d]Bound water molecules (see text)

[e]From KOH used for pH adjust

Table *S*2. SIMPSON calculations for possible distance pairs (^{2}H-^{19}F) to fit the experimental data (see Figure *S*6)

SIMPSON	REDOR distances, Å		RMSD[a]
	Short	Long	
Fit1	5.9	5.9	0.01
Fit2	5.6	6.2	0.01
Fit3	5.3	7.7	0.01
Fit4	5.0	7.7	0.01
Fit5	4.7	7.4	0.02

[a]root-mean-square deviation from the experimental data

Figure *S*1. Lee *et al.*

(A) Synthesis of compound **25**

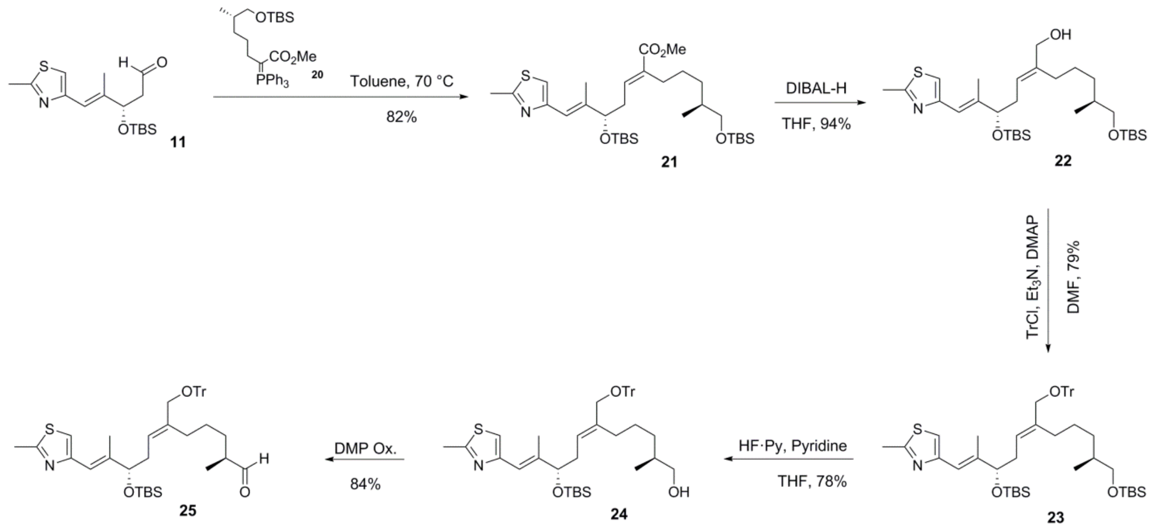

(B) Synthesis of compound **39**

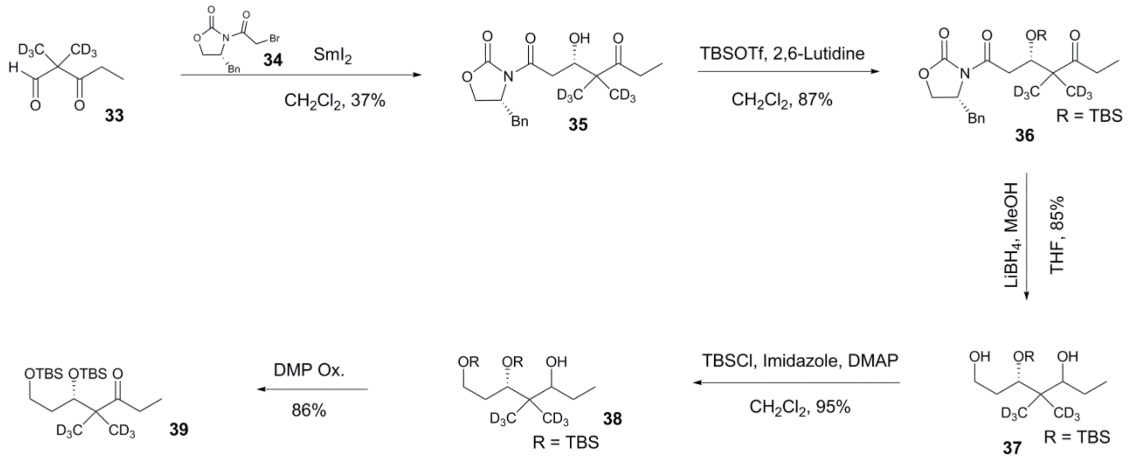

(C) Synthesis of compound **11**

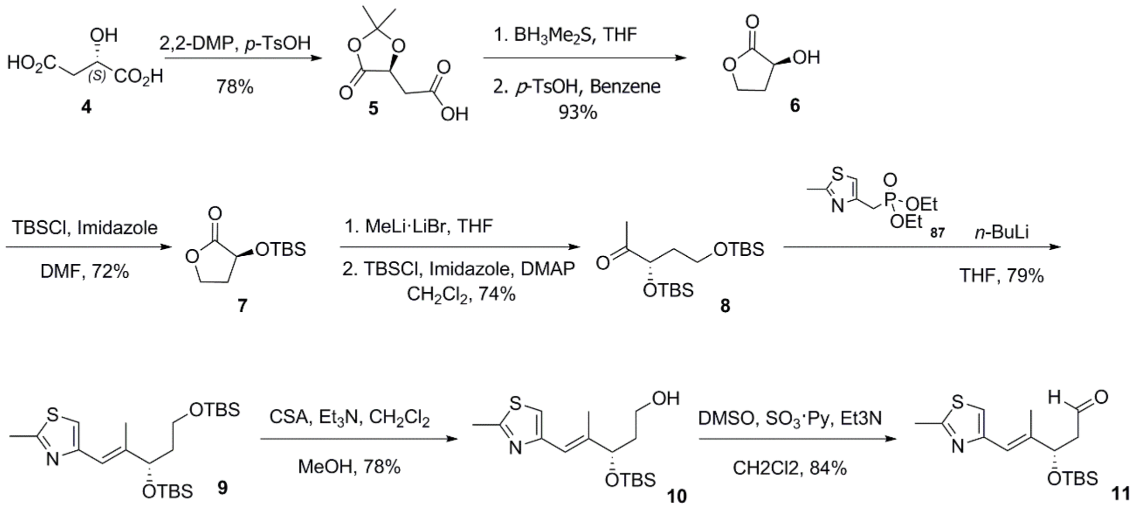

(D) Synthesis of compound **33**

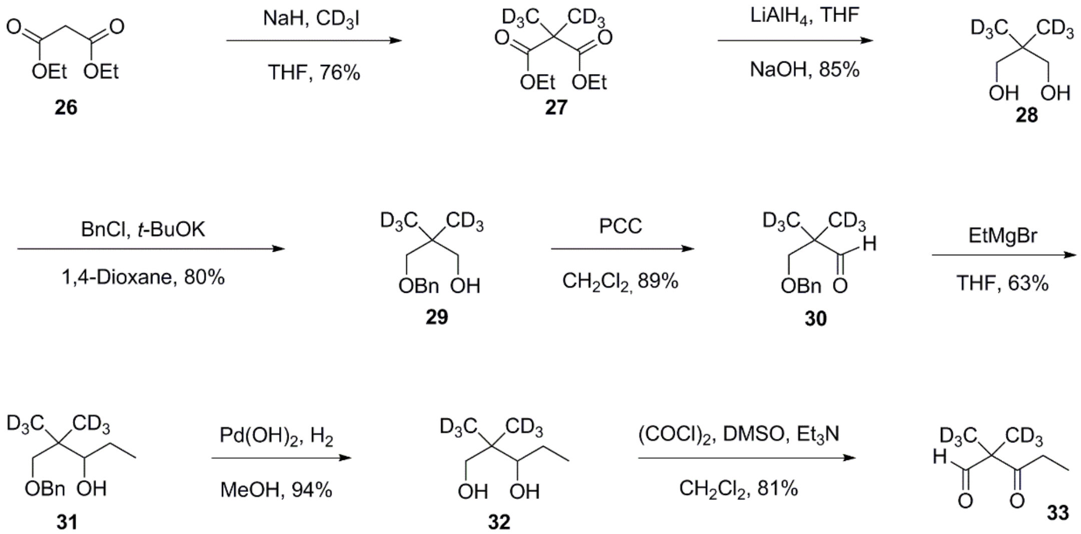

(E) Synthesis of compound **47**

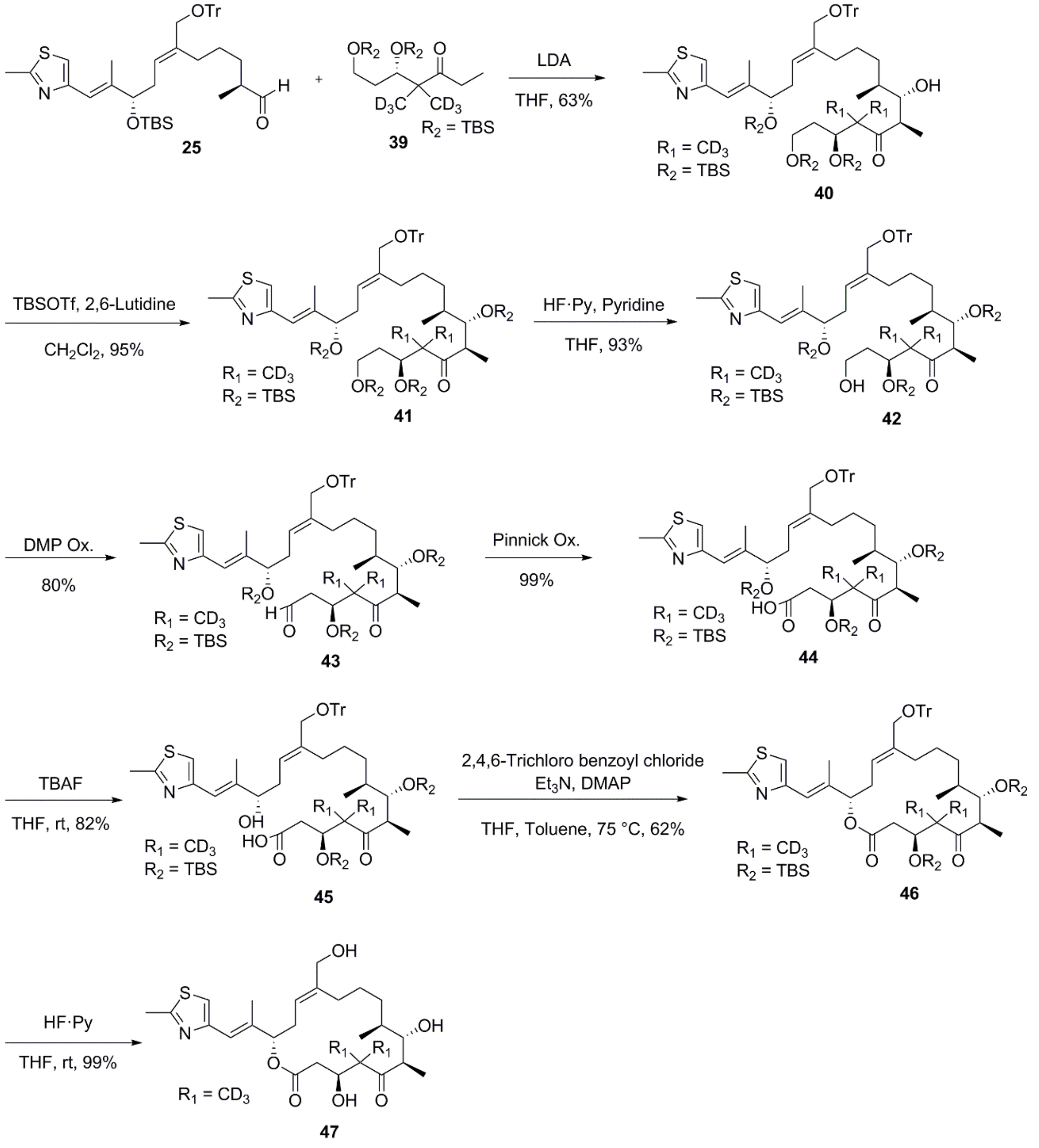

(F) Synthesis of compound **3**

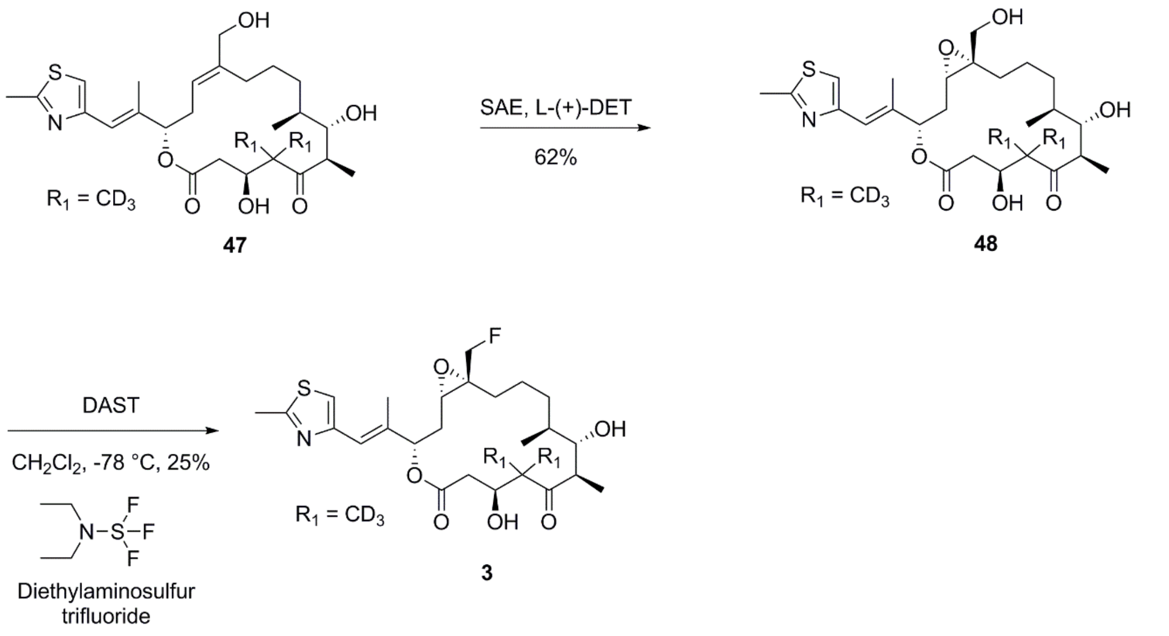

(G) Synthesis of compound **20**

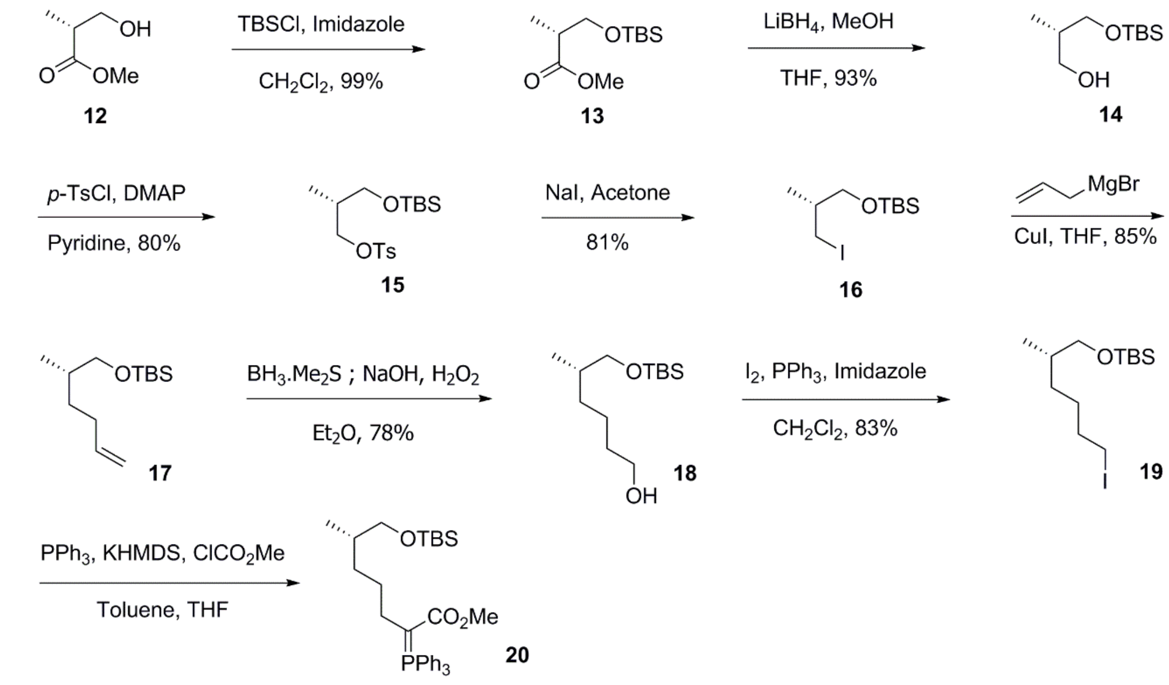

Figure *S*2. Lee *et al.*

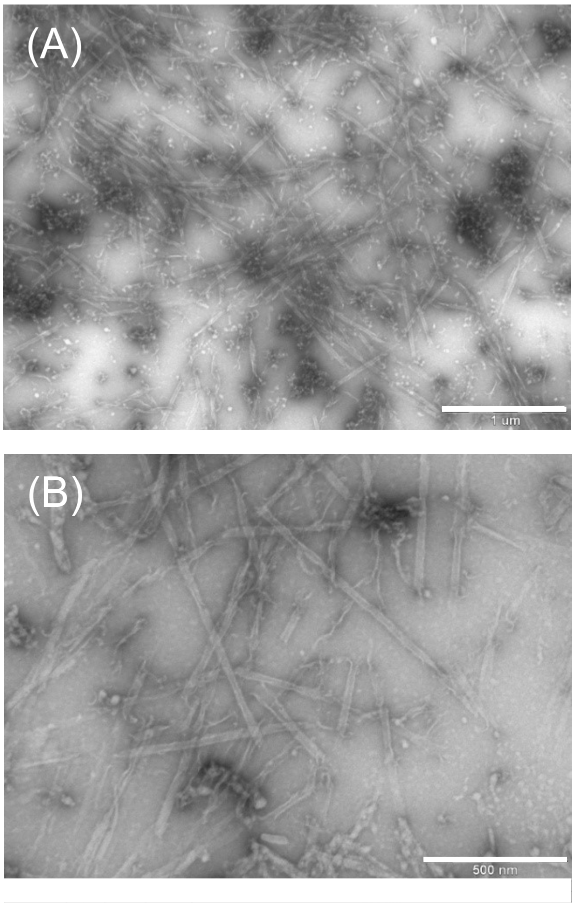

Fig. *S*3 Lee *et al.*

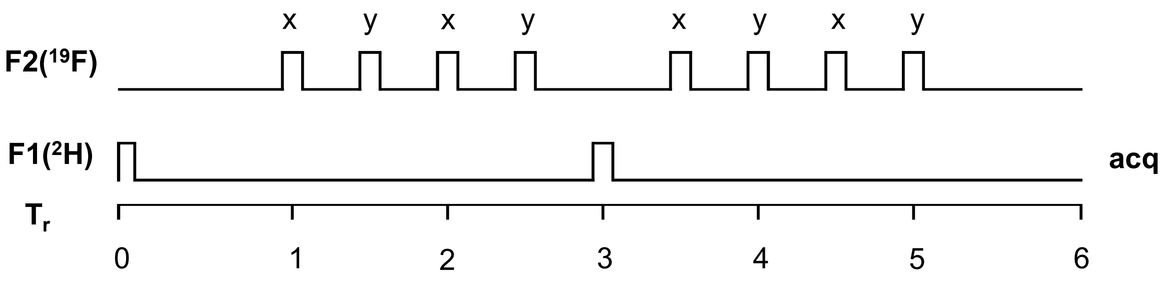

Figure *S*4. Lee *et al*.

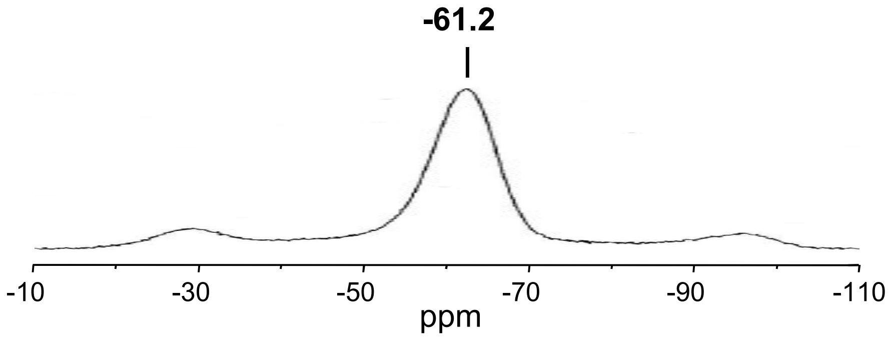

Figure *S5*. Lee *et al.*

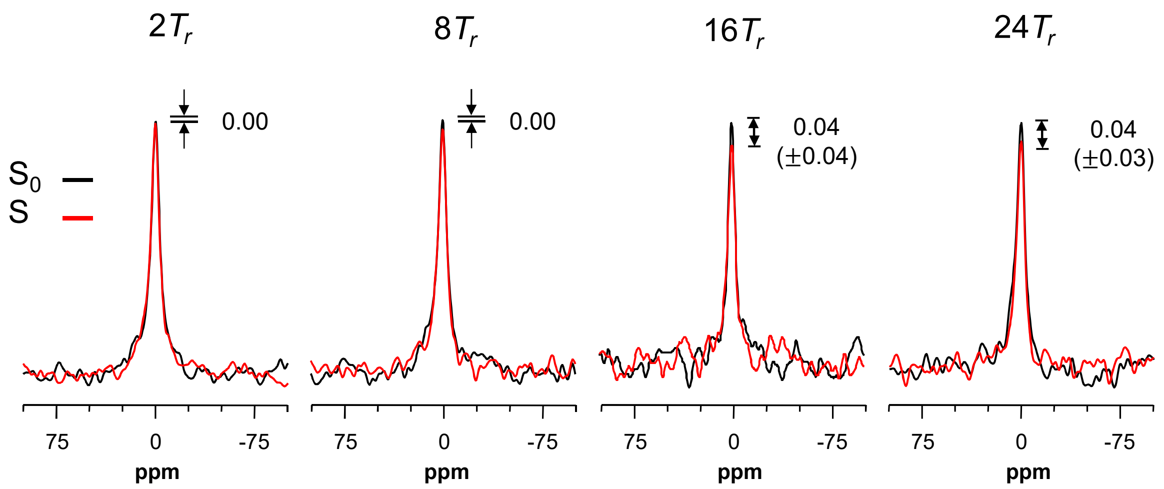

Figure *S*6. Lee *et al*.

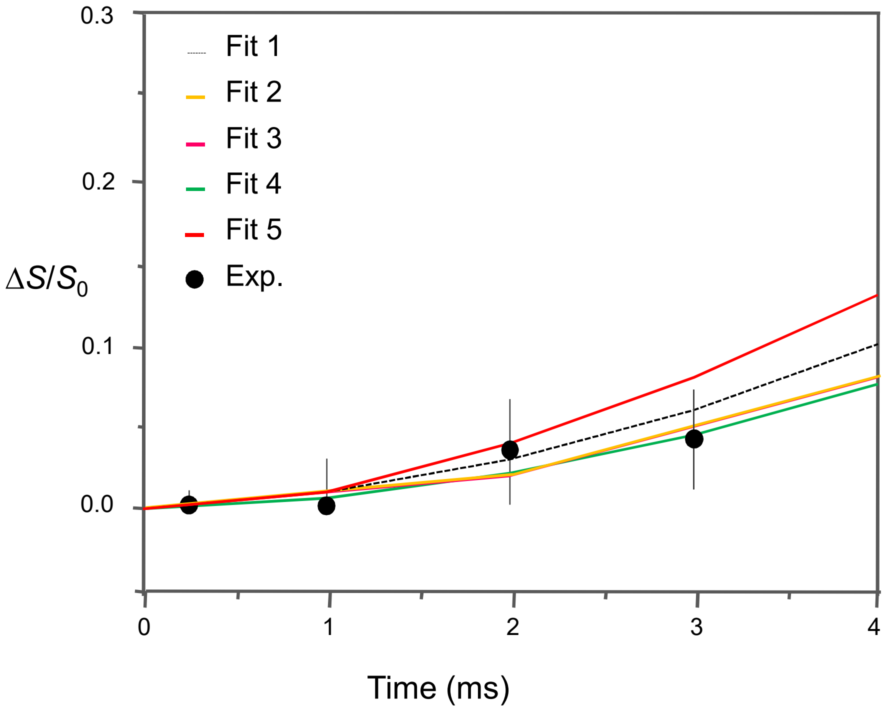

Figure *S*7. Lee *et al*.

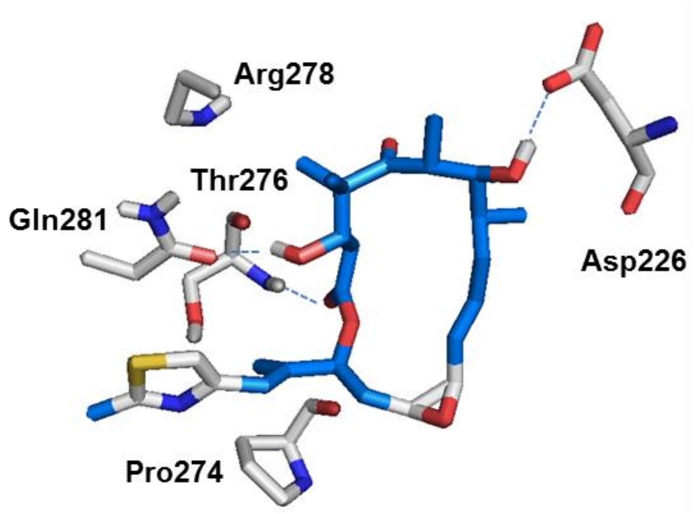

Figure *S*8. Lee *et al*.

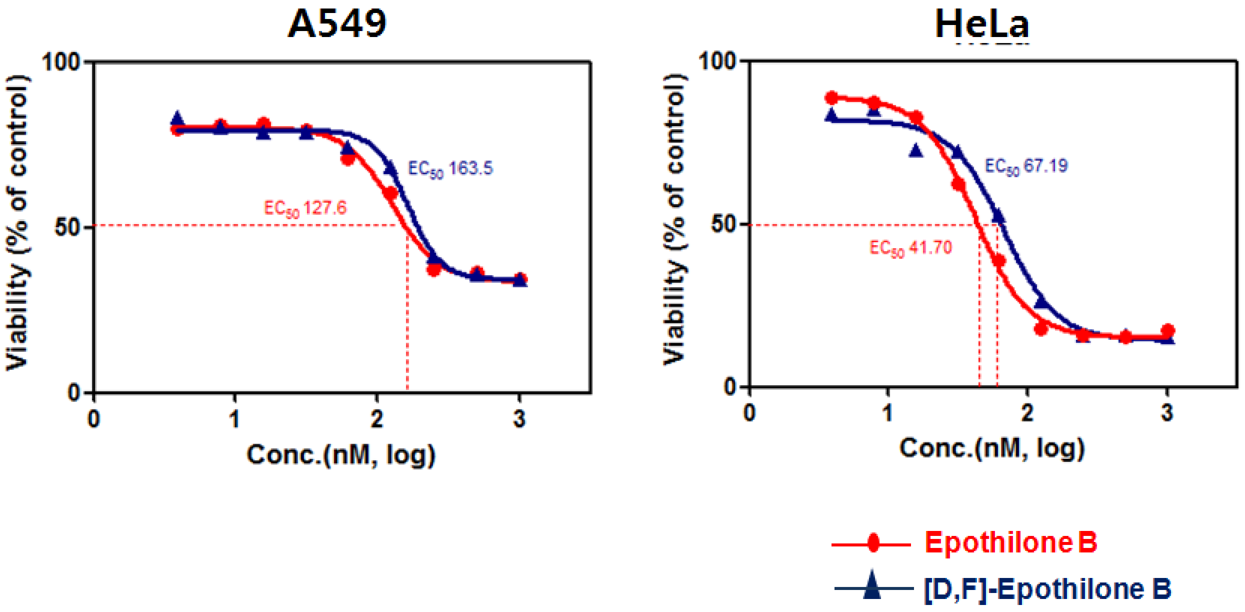

Supplement: Supplementary file 1 [file ijms-18-01472-s001.pdf]
